# Exploring Small RNA Function

**DOI:** 10.1371/journal.pbio.0020114

**Published:** 2004-02-24

Regulation of gene expression—deciding how much of what proteins are produced in the cell—is controlled by a myriad of different molecules. One type of naturally occurring regulatory molecule is small interfering RNA (siRNA), which selectively disrupts the production of a protein it is programmed to recognize, a process called RNA interference. These short stretches of nucleotides combine with other cellular proteins to form an RNA-induced silencing complex, called RISC, which locates and destroys a targeted messenger RNA—the molecule that carries a protein recipe from the nucleus to the site of production in the cytoplasm. While RNA interference has been widely exploited by researchers as a tool to knock out gene expression and infer the function of missing proteins, very little is known about the mechanisms behind this regulatory process.

Recently, biologists have discovered hundreds of other short pieces of regulatory RNA, called microRNAs, in both plants and animals. Like siRNA, they also affect gene expression, through similar, possibly even identical RISC molecules. Animal microRNAs, however, target messenger RNA at a different stage in protein production. Though researchers have determined the sequences of these microRNAs, uncovering their function—that is, which protein they interrupt and, in turn, what the interrupted protein does—has progressed slowly and sporadically without any decisive tool to study them. Only four animal microRNAs have known biological functions, despite the intense level of work going on in this field.

In this issue, György Hutvágner and colleagues report a rapid and reliable method for knocking out both siRNAs and microRNAs and thereby exploring their functions. The authors found that a short stretch of nucleotides, called a 2′-*O*-methyl oligonucleotide, whose sequence mirrors the targeted siRNA or microRNA, could bind and inhibit their function, allowing researchers an unprecedented glimpse at the regulatory roles and mechanisms behind RNA interference.

The authors first tested their oligonucleotide design against an siRNA known to interfere with production of the firefly protein, luciferase—this luminescent protein is often used as a “reporter,” lighting up when cells successfully produce the protein. Any interference means the glow is gone. Using extracts from fruitfly embryos as the test-bed, the researchers mixed in the luciferase-associated siRNA and the sequence-specific oligonucleotide. What holds these two molecules together is complementary base-pairing, the same force that holds two molecules of DNA together. As predicted, the oligonucleotide inhibited RISC activity—it could no longer silence the production of luciferase.

Because the authors could easily control the concentration of both the siRNA and the oligonucleotide inhibitor in these fly extract experiments, they were able to answer several questions about how these two molecules interact. They found that adding greater and greater concentrations of siRNA molecules did not result in equally great numbers of RISC; the process became saturated, indicating that a protein in the RISC assembly pathway limits production. Furthermore, the authors saw a marked 1:1 relationship between the concentration of the oligonucleotide and the concentration of RISC, indicating that each inhibitor binds to one RISC molecule in order to inactivate, a binding that appears to be irreversible. The results also showed that, though RISC molecules bind to the inhibitor through complementary base-pairing, a very different and more complex interaction is used by RISC molecules to find and bind their natural interference targets.

The authors then went on to use the luciferase siRNA to test the function of their oligonucleotide inhibitor in cultured human cells, which had been engineered to contain the luciferase gene. This in vivo experiment, using living and metabolizing cells, showed results similar to those with fruitfly extracts. But the real test for these inhibitors was to use them in a whole animal against a previously identified microRNA where the outcome of its inactivation was already known.

Hutvágner and colleagues constructed an oligonucleotide inhibitor based on the sequence of a microRNA called *let-7*, which blocks the production of the protein Lin-41 and is important for proper developmental timing in roundworm larvae. Larvae injected with the oligonucleotide had the exact features of a *let-7* deficient worm, showing that the inhibitor did indeed block this microRNA's function. The authors also used the oligonucleotides to provide evidence that two proteins, previously suggested to be involved with *let-7*, were directly associated with its interfering activity.

Using the technique described here, scientists could make rapid headway toward uncovering the biological functions of hundreds of microRNAs, their accessory RISC proteins, and even the proteins and genes they are programmed to interrupt. Furthermore, finding that RISC production is saturable could have significant implications for genetic studies that use RNA interference to uncover the function of sequenced, but unknown, genes; knowing the minimum required concentration of siRNA, researchers can avoid a buildup and any unwanted cell activity that goes along with it.

